# 4-*N*,*N*-Dimethylaminopyridine Promoted Selective Oxidation of Methyl Aromatics with Molecular Oxygen

**DOI:** 10.3390/molecules17043957

**Published:** 2012-03-30

**Authors:** Zhan Zhang, Jin Gao, Feng Wang, Jie Xu

**Affiliations:** 1 State Key Laboratory of Catalysis, Dalian National Laboratory for Clean Energy, Dalian Institute of Chemical Physics, Chinese Academy of Sciences, Dalian 116023, China; Email: zhangzhan@dicp.ac.cn (Z.Z.); gaojin@dicp.ac.cn (J.G.); wangfeng@dicp.ac.cn (F.W.); 2 Graduate university of Chinese Academy of Sciences, Beijing 100049, China

**Keywords:** organocatalysis, selectiveoxidation, methylaromatics, molecularoxygen, 4-*N*,*N*-dimethylaminopyridine

## Abstract

4-*N*,*N*-Dimethylaminopyridine (DMAP) as catalyst in combination with benzyl bromide was developed for the selective oxidation of methyl aromatics. DMAP exhibited higher catalytic activity than other pyridine analogues, such as 4-carboxypyridine, 4-cyanopyridine and pyridine. The sp^3^ hybrid carbon-hydrogen (C–H) bonds of different methyl aromatics were successfully oxygenated with molecular oxygen. The real catalyst is due to the formation of a pyridine onium salt from the bromide and DMAP. The onium salt was well characterized by NMR and the reaction mechanism was discussed.

## 1. Introduction

Selective oxidation of methyl aromatics to oxygenated aromatic chemicals is a fundamental process in the chemical industry. The corresponding carbonyl products are versatile building blocks in plastics, synthetic fibers, the pharmaceutical and perfume industries [1,2,3]. Traditionally, stoichiometric oxidants, such as KMnO_4_ and HNO_3_, were used for the oxidation, which leads to large amounts of chemical waste [3]. In contrast, the catalytic oxidation using molecular oxygen as terminal oxidant is attractive from both the economical and environmental viewpoints [4]. 

Many catalytic systems have been developed for the catalytic oxidation of methyl aromatics with molecular oxygen, most of which involve metal species [2,5,6]. However, traces of toxic metal ions could contaminate the products, and metal catalysts subject to deactivation [7,8,9]. Organocatalysts which exhibit good performance have attracted much attention, and is an area that has rapidly developed recently [10]. Encouraging advances have been reported in the field of metal-free oxidation with organocatalysts. For example, 10-methyl-9-phenylacridinium ion derivatives were developed for the highly selective oxygenation of *p*-xylene [11,12]. 2-Chloroanthroquinones were also reported as efficient organocatalysts for the oxidation of methyl aromatics under mild conditions [13]. These processes were performed under light irradiation. Our group has focused on the catalytic system consisting of the *N*-hydroxyphthalimide (NHPI) analogues and different organic mediators for catalytic oxidation of hydrocarbons with molecular oxygen [1,14,15,16,17,18,19]. All these works demonstrated the great potential of organocatalytic systems for metal-free oxidation with molecular oxygen.

Bromo species were efficient for activating C–H bonds in the oxidation of methyl aromatics. The most widely used commercial catalytic package for industrial oxidation of methyl aromatics usually combines cobalt acetate, manganese acetate and a bromide source (e.g., HBr, NaBr) [20]. However, transition metals are generally indispensable to catalysis. It was reported that bromo sources such as allylbromide and CBr_4_ could catalyze metal-free oxygenation with molecular oxygen under photo-irradiation [21,22]. We recently utilized a catalytic amount of bromine for the metal-free oxidation of various aromatic hydrocarbons promoted by nitrogen-containing compounds (e.g., 1, 10-phenanthraline, acridine yellow, methyl violet) in acetonitrile [23,24,25]. Now catalysis by 4-*N*,*N*-dimethylaminopyridine (DMAP) in combination with benzyl bromide has been developed for the same purpose. DMAP exhibited higher catalytic activity than other pyridine analogues, such as 4-carboxypyridine, 4-cyanopyridine and pyridine.

## 2. Results and Discussion

### 2.1. Catalytic Oxidation of *p*-xylene with Molecular Oxygen Using DMAP/Benzyl Bromide

**Scheme 1:**
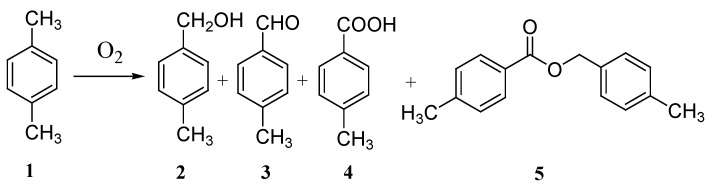
Catalytic oxidation of *p*-xylene.

Initially solvent-free oxidation of *p*-xylene was selected as the model reaction (Scheme 1). The results are listed in Table 1. Few oxygenated products were detected without any catalyst or in the presence of DMAP (Table 1, entries 1–2). When benzyl bromide was used, only 6% conversion of *p*-xylene was obtained (Table 1, entry 3), which indicated benzyl bromide was a catalytically inert bromo source [26,27]. When DMAP was employed in combination with benzyl bromide (Table 1, entry 4), the *p*-xylene conversion increased substantially to 49% under the same reaction conditions. It is proposed that DMAP and benzyl bromide have a synergistic effect in the catalysis. They exhibited different characteristics from metal catalysts. For the reaction in the presence of DMAP and benzyl bromide, the major product was *p*-toluic acid (**4**) with a selectivity of 58%; the selectivities for *p*-tolyl alcohol (**2**) and *p*-tolualdehyde (**3**) were 7% and 13%, respectively; and the selectivity for 4-methylbenzyl 4-toluate (**5**) was 19%. In metal catalyzed oxidations of *p*-xylene, *p*-toluic acid or terephthalic acid were usually obtained as the major products [8,20]. The primary oxidation products such as **2** and **3** were easily further oxidized, and product **5** was usually formed with low selectivity. 

**Table 1:** Oxidation of *p*-xylene under solvent-free conditions ^[a]^.

Entry	Catalyst	Conversion(%)	Product selectivity (%)				
			2	3	4	5	Others
1	None	<2	-	-	-	-	-
2	DMAP	<2	-	-	-	-	-
3	benzyl bromide	6	50	50	n.d. ^[b]^	n.d.	n.d.
4	DMAP/benzyl bromide ^[c]^	49	7	13	58	19	3

^[a]^ Reaction conditions: *p*-xylene (100 mmol), catalyst (2.5 mmol), 1.0 MPa O_2_, 160 °C, 3 h; ^[b]^ n.d., not detected; ^[c]^ 2.5 mmol of DMAP and 2.5 mmol of benzyl bromide were used.

### 2.2. Influence of DMAP/Benzyl Bromide Molar Ratio

**Figure 1:**
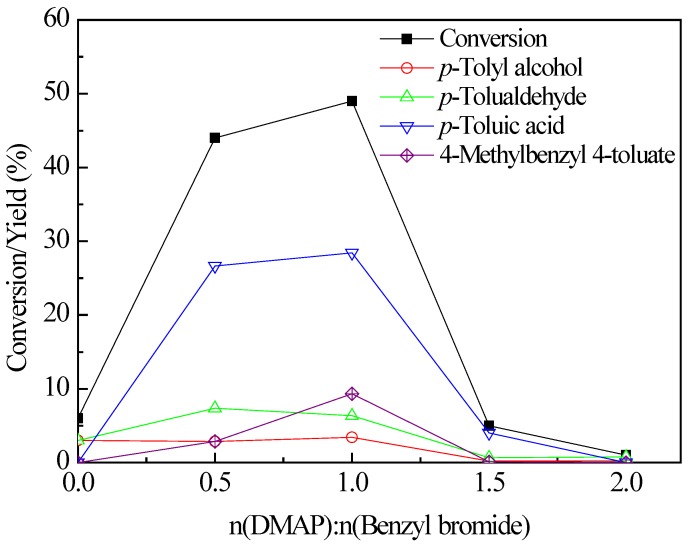
Influence of DMAP/benzyl bromide molar ratio; reaction conditions: 100 mmol of *p*-xylene, 2.5 mmol of benzyl bromide, 160 °C, 1.0 MPa O_2_, 3 h.

The molar ratio of DMAP/benzyl bromide has great effect on the oxidation (Figure 1). Decreasing n(DMAP):n(benzyl bromide) from 1.0 to 0.5 slightly reduced the *p*-xylene conversion from 49% to 41%. 

However, when the n(DMAP):n(benzyl bromide) ratio was increased to 1.5, the *p*-xylene conversion was sharply reduced to 5%, and an even higher ratio of n(DMAP):n(benzyl bromide) = 2 resulted in no activity. Previous studies have reported amine bases could work as anti-oxidants [8,26]. Therefore, excess amount of DMAP probably inhibited the oxidation process.

### 2.2. Influence of Different Pyridine Analogues

**Figure 2:**
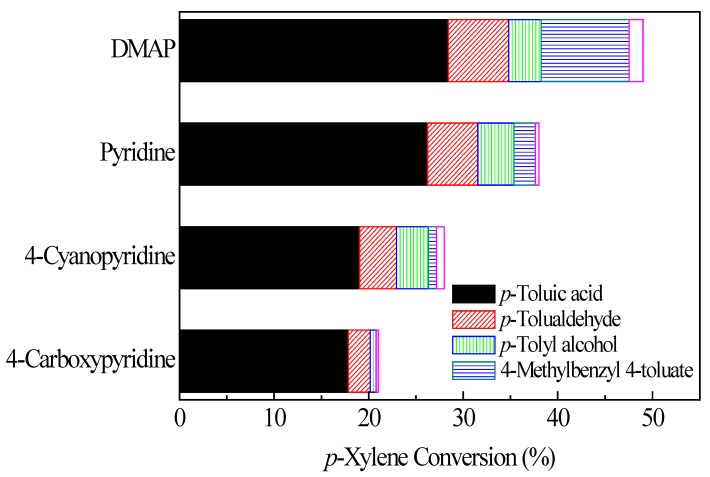
*p*-Xylene oxidation with different pyridine analogues; reaction conditions: *p*-Xylene (100 mmol), benzyl bromide (2.5 mmol), pyridine analogues (2.5 mmol), 1.0 MPa O_2_, 160 °C, 3 h.

Encouraged by the results, we further tested several different pyridine analogues for catalytic oxidation of* p*-xylene (Figure 2). DMAP showed better catalytic activity than 4-carboxypyridine, 4-cyanopyridine and pyridine. The conversion of *p*-xylene increased in the order of 4-carboxy-pyridine < 4-cyanopyridine < pyridine < DMAP. Electron-donating group substituted pyridine exhibited higher activity than electron-withdrawing group substituted pyridine.

### 2.3. Optimization of Reaction Conditions

**Figure 3:**
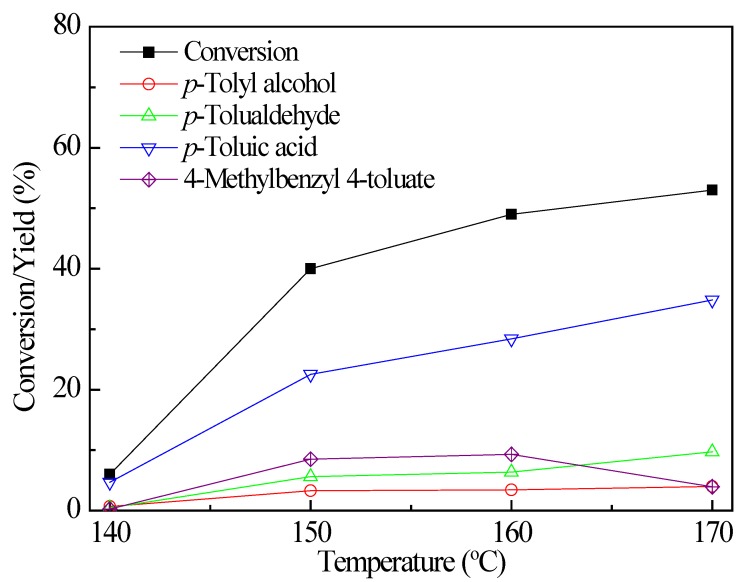
The influence of temperature; reaction conditions: *p*-Xylene (100 mmol), DMAP (2.5 mmol), benzyl bromide (2.5 mmol), 1.0 MPa O_2_, 3 h.

More detailed optimization of the reaction conditions including temperature and time was also carried out. The influence of temperature on the oxidation of *p*-xylene is shown in Figure 3. When the temperature was increased from 160 °C to 170 °C, the *p*-xylene conversion increased a little from 49% to 53%, accompanied by an increase in the by-products including 2-benzyl-*p*-xylene and 2,5,4'-trimethyldiphenylmethane. The formation of the by-products could be caused by alkylation of *p*-xylene [28,29]. The decreased reaction temperature led to much lower conversion. Therefore, the reaction temperature was optimized as 160 °C.

**Figure 4:**
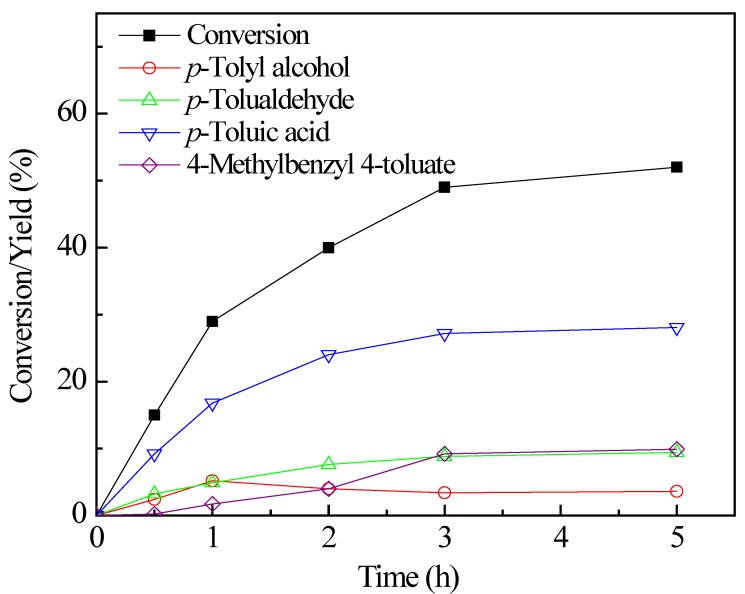
The influence of time; reaction conditions: *p*-Xylene (100 mmol), DMAP (2.5 mmol), benzyl bromide (2.5 mmol), 1.0 MPa O_2_, 160 °C.

The time-on-course of *p*-xylene oxidation is shown in Figure 4. The *p*-xylene conversion increased steadily along the reaction in first 3 h. After 3 h there was no remarkable increase in *p*-xylene conversion by prolonging the reaction time. In the solvent-free oxidation, it was noticed that the solubility of the oxidation product of *p*-toluic acid **4** is low. The increasing amount of the solid product **4** may hinder the mass transfer for the oxidation process. 

In order to improve the catalytic results, we investigated the effect of the different solvents listed in Table 2. Using water as the solvent, the oxidation of *p*-xylene was inhibited (Table 2, entry 1). Acetic acid is a good solvent for many oxidation reactions [8,30], but a reaction between acetic acid and the oxidation intermediate occurred in this case, which led to the formation of 4-methylbenzyl acetate (Table 2, entry 2). In acetonitrile, a *p*-xylene conversion of 87% was obtained with a selectivity of 82% for *p*-toluic acid **4** (Table 2, entry 3). This improvement could be due to the improved mass transfer. We attempted to perform the reaction under lower temperatures, but the reaction was still sluggish (Table 2, entries 4 and 5). Despite the fact the conversion of *p*-xylene was improved in solvents, several points should be considered. On the one hand, the loss of solvent cannot be neglected under the reaction conditions. On the other hand, the neat reaction may give a clear reaction result with less interference for understanding the oxidation process. Therefore, the subsequent experiments were still conducted in the absence of solvent.

**Table 2:** Oxidation of *p*-xylene in different solvents ^[a]^.

Entry	Solvent	Temperature (°C)	Conversion(%)	Product selectivity (%)			
				3	4	5	Others ^[b]^
1	H_2_O	160	<2	-	-	-	-
2	Acetic acid	160	66	17	60	1	22 ^[c]^
3	Acetonitrile	160	87	7	82	2	9
4	Acetonitrile	140	28	31	67	-	2
5	Acetonitrile	120	<2	-	-	-	-

^[a]^ Reaction conditions: *p*-xylene (10 mmol), benzyl bromide (0.25 mmol), DMAP (0.25 mmol), solvent (5 mL), 1.0 MPa O_2_, 160 °C, 3 h; ^[b]^ Mainly 4-carboxylic benzaldehyde and terephthalic acid; ^[c]^ Mainly 4-methylbenzyl acetate.

### 2.4. Oxidation of Different Methyl Aromatics

To investigate the scope and limitation of our catalytic system, the oxidation of several different methyl aromatics were carried out (Table 3). As can be seen, the use of this catalytic system could be extended to different methyl aromatics. Relative high conversions were obtained for xylenes, 1,3,5-trimethylbenzene and *p*-*tert*-butyltoluene (Table 3, entries 1–4). This should be attributed to the activation of the methyl group by those electron donating substitutent groups. Without this substitution effect, toluene, *p*-chlorotoluene, *p*-bromotoluene and 1-methylnaphthalene were less easily oxidized, even at elevated temperature (Table 3, entries 5–8).

**Table 3:** Oxidation of different methyl aromatics ^[a]^.

Entry	Substrate	Conversion(%)	Product selectivity (%) ^[b]^				
			Alcohol	Aldehyde	Acid	Ester	Others
1	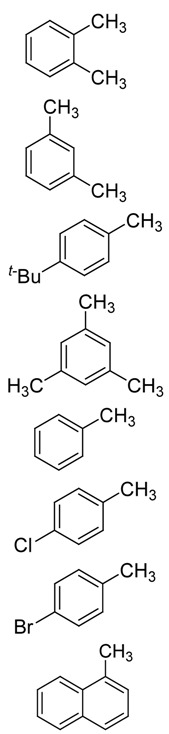	56	5	8	75	10	2
2		41	3	4	82	10	1
3		49	3	6	89	1	1
4		39	2	7	86	4	1
5 ^[c]^		17	10	16	58	14	2
6 ^[c]^		14	6	18	56	17	3
7 ^[c]^		12	7	17	63	11	2
8 ^[c]^		<2	-	-	-	-	-

^[a]^ Reaction conditions: substrate (100 mmol), DMAP (2.5 mmol), benzyl bromide (2.5 mmol), 160 °C, 1.0 MPa O_2_, 3 h; ^[b]^ For multimethyl aromatics, only monomethyl oxygenated products were detected as the main products; ^[c]^ Reaction was carried out at 180 °C.

### 2.5. Interaction of DMAP and Benzyl Bromide

In order to better understand the reaction, control experiments were carried out (Table 4). When benzyl chloride was used instead of benzyl bromide, the conversion of *p*-xylene was only 2%, which was much lower than that seen with the usage of benzyl bromide (Table 4, entry 1 and Table 1, entry 4). This result indicated that bromo species was crucial for the catalysis. Different combinations of bromo-containing chemicals with DMAP were compared for the oxidation (Table 4, entries 2–5). With molecular bromine, only 6% of *p*-xylene was converted and bromination of the aromatic ring was observed. Ammonium bromide was also less efficient, with a conversion of only 20%. In contrast, when alkyl bromides such as *n*-butylbromide and *n*-dodecylbromide were employed, the *p*-xylene conversion reached 45% and 44%, respectively, which was slightly lower than that seen when benzyl bromide was used. The alkyl bromides were preferred in combination with DMAP for solvent-free oxidation of *p*-xylene.

**Table 4:** Comparison of different bromide sources for *p*-xylene oxidation ^[a]^.

Entry	Catalysts	Conversion(%)	Product selectivity (%)				
			2	3	4	5	Others
1	benzyl chloride + DMAP	2	16	84	n.d. ^[b]^	n.d.	n.d.
2	bromine + DMAP	6	27	43	n.d.	n.d.	30^[c]^
3	ammonium bromide + DMAP	20	20	19	39	19	3
4	*n*-butyl bromide + DMAP	45	5	8	65	20	2
5	*n*-dodecyl bromide + DMAP	44	9	11	58	21	1
6	1-benzyl-4-*N*,*N*-dimethyl pyridinium bromide ^[d]^	51	6	15	59	17	3

^[a]^ Reaction conditions: *p*-Xylene (100 mmol), DMAP (2.5 mmol), bromo-containing chemical (2.5 mmol), 160 °C, 1.0 MPa O_2_, 3 h; ^[b]^ n.d., not detected; ^[c] ^Bromination products were determined as the main byproducts; ^[d]^ 2.5 mmol of pyridinium onium salt was used as catalyst.

**Figure 5:**
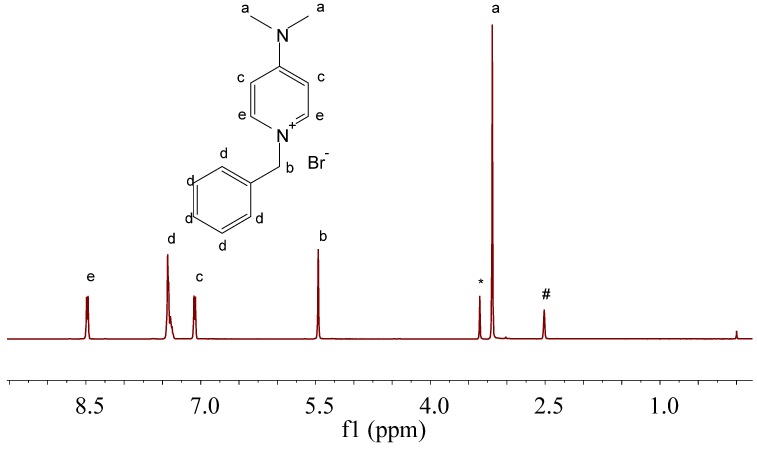
^1^H-NMR of the separated pyridine onium salt; the signal at 2.51 ppm is attributed to DMSO solvent residue, and that at 3.35 ppm is attributed to water existed in DMSO-*d_6_*.

For oxidations of methyl aromatics involving bromo species, benzyl bromide (or its analogues) are easily formed, and are usually thought to be inert for catalytic oxidation [26,27]. When DMAP was introduced, benzyl bromide readily reacted with this nucleophlic base and formed the corresponding onium salt. Previous reports have noticed that the onium salts were effective in promoting hydrocarbon oxidations [31,32]. Therefore, the *in situ *formation of a pyridine onium salt may account for the enhanced activity of DMAP/benzyl bromide. Indeed, when DMAP and benzyl bromide were stirred under 160 °C in *p*-xylene, the pyridine onium salt can be separated as a pure product. The structure was confirmed by ^1^H-NMR as 1-benzyl-4-*N,N*-dimethylpyridinium bromide [δ = 8.48 (d, 2H), 7.55–7.29 (m, 5H), 7.12–7.04 (m, 2H), 5.51–5.42 (m, 2H), 3.24–3.14 (m, 6H); Figure 5]. Using this onium salt as catalyst, the *p*-xylene oxidation proceeded with a conversion of 51% (Table 3, entry 6), which is comparable to the result employing DMAP and benzyl bromide as shown in Table 1, indicating that the real catalyst could be the corresponding pyridine onium salt. 

### 2.6. Tentative Reaction Mechanism

**Scheme 2:**
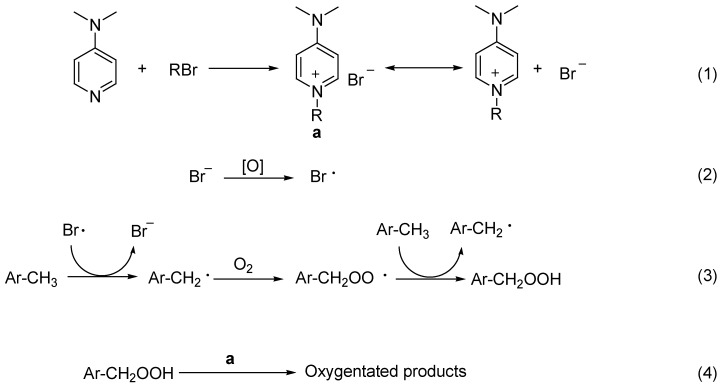
Tentative reaction route.

The liquid-phase oxidation of hydrocarbon generally follows the radical chain oxidation mechanism. The present reaction may be also based on the same mechanism, which is supported by the observed inhibition of oxidation in the presence of trace amount of *p*-cresol. In light of all information above, we propose the reaction mechanism to be as shown in Scheme 2. Alkyl bromide readily reacts with DMAP and forms the corresponding onium salt. The organic bromide is converted into ionic bromide, which is easily oxidized into a bromo radical under elevated temperature [8,33]. The bromo radical then abstracts hydrogen atom from the benzylic positions. Similar activation of hydrocarbons by a bromo source is observed in photooxidations [21,22]. This may explain why the bromo source is crucial for the catalysis. The benzyl radical traps molecular oxygen and forms a peroxy radical and hydroperoxide. According to previous reports, the decomposition of hydrperoxide can be promoted by onium salts, which is favorable for further propagation of the radical chain oxidation and affords oxygenated products [31,32]. 

## 3. Experimental

### 3.1. General

All reagents and materials were commercially available products purchased from J&K Chemical Co. Ltd. and Tianjin Kermel Chemical Reagent Co. Ltd., China. Different methyl aromatics were used after removing water by adding 5 wt% of 4Å molecular sieves and treating for 24 h. Other reagents were directly used without further purification. The conversion of methyl aromatics and selectivity of products were determined using an Agilent-7890 GC equipped with a FID and using 1,2-dichlorobenzene as the internal standard. The products were identified on an Agilent 6890N GC/5973 mass spectrometer (MS). The ^1^H-NMR spectra were recorded in DMSO-*d*_6_ on a Bruker 400 MHz NMR spectrometer with TMS as internal standard.

The reactions were carried out in a closed Teflon-lined 100 mL autoclave equipped with a magnetic stirrer and an automatic temperature controller. In a typical experiment, *p*-xylene (100 mmol) were added into the autoclave followed by DMAP (2.5 mmol) and benzyl bromide (2.5 mmol). After the autoclave was sealed, the air inside was purged and replaced with O_2_ three times, then the autoclave was heated to 160 °C. The pressure was kept at 1.0 MPa during the reaction by continuously recharging oxygen as needed. After 3 h the autoclave was cooled to room temperature and carefully depressurized. Dimethyl formamide (DMF, 10 mL) was used to dissolve the products before analysis.

## 4. Conclusions

In summary, a metal-free catalytic system consisting of DMAP and benzyl bromide was developed. The primary carbon-hydrogen bonds in different methyl aromatics could be oxidized with molecular oxygen using this catalyst. The major products are single-substituted organic acids. The* in situ* formation of a pyridine onium salt via reaction of DMAP and benzyl bromide may function as the real catalyst in the reaction. Our study contributes to the basic understanding of organocatalytic systems for metal-free oxidation. 
